# Genotype x environment interaction effect on grain yield of cowpea (*Vigna unguiculata* (L.) Walp) in Deciduous forest and Sudan savanna ecologies of Ghana

**DOI:** 10.1371/journal.pone.0314464

**Published:** 2025-01-24

**Authors:** Francis Kusi, Richard Adu Amoah, Patrick Attamah, Shaibu Alhassan, Damba Yahaya, Justice Frederick Awuku, Jerry A. Nbonyine, Isaac Amegbor, Gloria Mensah, Issah Sugri, Mukhtaru Zakaria, Salim Lamini, Peter Asungre, Emmanuel Asibi Aziiba, Julius Yirzagla, Emmanuel Boamah Duku, Daniel Ashie Kotey, Isaac Asante

**Affiliations:** 1 CSIR-Savanna Agricultural Research Institute, Tamale, Ghana; 2 CSIR-Plant Genetic Resources Research Institute, Bunso, Ghana; 3 University for Development Studies, Department of Biotechnology, Tamale, Ghana; 4 Department of Botany, University of Ghana, Legon, Ghana; Nepal Agricultural Research Council, NEPAL

## Abstract

Cowpea is deemed as a food security crop due to its ability to produce significant yields under conditions where other staples fail. Its resilience in harsh environments; such as drought, heat and marginal soils; along with its nitrogen-fixing capabilities and suitability as livestock feed make cowpea a preferred choice in many farming systems across sub-Saharan Africa (SSA). Despite its importance, Cowpea yields in farmers’ fields remain suboptimal, primarily due to biotic and abiotic factors and the use of either unimproved varieties or improved varieties that are not well-suited to local conditions. Multi environment testing of genotypes is essential for recommending varieties suited for either specific or for wide cultivation. This study aimed, to identify and recommend cowpea breeding lines for wide or specific cultivation in the Sudan Savanna and Deciduous Forest zones of Ghana. The research utilized twenty early-maturing advance cowpea breeding lines and three check varieties (released varieties). The experiment was conducted in two locations: Bunso in the Deciduous Forest zone and Manga in the Sudan Savanna zone over 2020/2021 and 2021/2022 cropping seasons. Combined analysis of variance revealed a significant genotype-environment interaction (GEI) which accounted for 35.12% of the variation in yield. The environments were classified into three mega environments, with Bunso_2021 identified as the near-ideal environment where the genotypes exhibited their maximum genetic potentials. In terms of adaption, genotype UG_04 demonstrated broad adaption, showing high yield and stability across all test environments. Genotypes UG_01 and UG_02 performed particularly well in Bunso_2021 and Bunso_2022, while UG_04 and UG_14 excelled in Manga_2021. These findings provide valuable insights for selecting cowpea varieties that can enhance productivity and stability in diverse agro-ecological zones.

## Introduction

Cowpea (*Vigna unguiculata* (L.) Walp) is a diploid (2n = 2x = 22) legume cultivated in sub-Saharan Africa for its protein rich grains [1]. It is the most cultivated and consumed legume after groundnut in the sub region. Cowpea plays a major role as the protein source in the diets of resource poor households, hence often referred to as “poor man’s meat” [2, 3]. Aside meeting the dietary needs, cowpea contributes to income generation, soil fertility amendments and fodder for livestock fatting of small holder farmers [1].

Despite having a potential yield of over 2 t/ha, the average yields of cowpea on farmers’ fields are below 0.6 t/ha [1]. This low yield is due to the use of unimproved varieties, biotic and abiotic stresses [4, 5]. Some of the abiotic stresses that affect cowpea production are drought, heat, flooding and soil salinity. The biotic stresses include insect pests and diseases, parasitic weeds such *Striga* and *Alectra* and nematodes. In an environment, variations such as location conditions, prevailing seasonal changes and the interplay of these factors affect the yield of cowpea on farmers’ fields [6].

Genotype-environment interaction (GEI) is the differential responses of genotypes across different environments. The phenomenon of genotype-environment interaction (GEI) is problematic to plant breeders since it obscures variety recommendation due to the discrepancy of best-yielding genotypes across cropping seasons and environments. Breeders therefore use genotype-environment interaction studies as a strategy for testing and selecting genotypes adapted to target environments [7]. Based on the outcome of GEI studies, genotypes may be identified for specific or wide adaptation. Genotypes with stable mean yields across environments are said to have wide adaptation. Genotypes with location specific adaptation on the other hand, are those that are high yielding in one particular environment but low yielding in other environments [8]. GEI studies are important because the performance of a genotype can be affected by its genetics, its environments, or the interaction of both [9, 10]. The genotypic potential of the genotype can be concealed by the environment, resulting in poor genetic gain from selection of quantitative traits [10].

In the studies of GEI, various methods have been used to analyze and interpret multi-environment trails data. These include the main genotype effect plus genotype-by-environment interaction biplot (GGE biplot) analysis and the additive main effect and multiplicative interaction (AMMI) analysis, Finlay–Wilkinson model and Eberhart and Russell model [10–12]. The effect of GEI is mostly reduced by trailing the genotypes at multilocation in a single year, or over several years in a single location, or both [13]. Multi-location evaluations give vital information about the adaptations and stability of advanced breeding lines prior to their release [14]. The effect of GEI is minimal when genotypes have wide adaptations [9] or when the area of cultivation is partitioned into mega-environments in which similar locations can be found [10].

In Ghana, cowpea is produced in all agro-ecological zones of the country under rainfed conditions. These zones differ in terms of rainfall, soil type and fertility, temperature and other conditions that affect the cultivation of crops. Cowpea is cultivated in Sudan Savanna zone from July to October while production in the deciduous forest agro-ecologies is carried out during the minor rainy season from August to December. Yield variability across various environments necessitate multi-location evaluation of improved cowpea genotypes to make recommendations for either specific or widespread cultivation. The objective of this study was therefore to evaluate advanced cowpea breeding lines for their adaptability and yield stabilities in the Sudan savanna and deciduous forest agro-ecologies of Ghana.

## Material and methods

### Site description and planting materials

The experiments were carried out in two locations in Ghana in 2021 and 2022 cropping seasons under rainfed conditions. The two locations were the research fields of the CSIR- Savanna Agriculture Research Institute, Manga in the Sudan Savanna and CSIR-Plant Genetic resources Research Institute, Bunso in the Deciduous Forest agro-ecological zone of Ghana (Table 1). The Sudan Savanna is characterized by a unimodal rainy season that spans from June to October and a dry season lasting from November to May. Regions of the Sudan Savanna are Striga endemic regions with *Striga gesnerioides* reducing the yield of cowpea. Bunso on the other hand has two rainy seasons, the major season from March to July and the minor rainy season from August to December. The conditions in Bunso during the 2021 season was considered as Environment 1 (E1), Bunso in 2022 as Environment 2 (E2), whereas Manga in 2021 season was Environment 3 (E3) and Manga 2022 as Environment 4 (E4). The soil pH, textural classes and nutrient levels of the experimental sites are presented in Table 2.

**Table 1:** Summary description of four test environments for testing 23 cowpea genotypes.

Environment	Vegetation type	Rainfall	Geographical location		Temperature (°C)	
			Lat. (N)	Long. (E)	Min.	Max.
E1	Semi-deciduous forest	1743.70 mm	6°17’46" N	0°27’39" W	29.37	31.03
E2	Semi-deciduous forest	1356.60 mm	6°17’40" N	0°27’36" W	26.53	28.30
E3	Sudan Savanna	877.75 mm	11°0’58" N	0°15’58" W	32.02	34.2
E4	Sudan Savanna	1144.73 mm	11°0’54" N	0°15’59" W	30.20	33.1

Bunso in 2021 as Environment 1 (E1), Bunso in 2022 as Environment 2 (E2), whereas Manga in 2021 season was Environment 3 (E3) and Manga 2022 as Environment 4 (E4).

**Table 2:** Soil properties of the experimental sites.

Environment	pH	Textural Class	Phosphorus mg/kg	% Total Nitrogen	Potassium (cmol/kg)	Calcium (cmol/kg)	Magnesium (cmol/kg)	% Organic matter
E1	6.85	Loamy sand	10.23	0.56	2.09	17.6	5.8	1.86
E2	6.48	Loamy sand	8.97	0.61	2.5	14.5	5.9	1.7
E3	5.6	Sandy	1.4	0.03	24	0.9	0.8	0.5
E4	5.9	Sandy	2.6	0.06	20	0.75	1.2	0.95

Twenty early maturing advance cowpea breeding lines and 3 checks (release varieties) were used in the study. The 20 breeding lines were developed by University of Ghana while the checks were developed and released by CSIR-Savanna Agricultural Research Institute (CSIR-SARI). The checks are high yielding, early maturing varieties that are mostly cultivated by farmers. They have white seed coat colour which is preferred by consumers. Two checks, Wang Kae and Kirkhouse Benga are resistant to aphid and striga which are serious constraints of cowpea production. Apagbaala on the other hand is susceptible to aphids and *striga gesnoroides*. The list of the genotypes and their description are presented in Table 3.

**Table 3:** List of cowpea genotypes and their sources.

Genotype code	Genotypes	Status	Year of release	Source
1	UG_01	Advanced breeding line	-	University of Ghana
2	UG_02	Advanced breeding line	-	University of Ghana
3	UG_03	Advanced breeding line	-	University of Ghana
4	UG_04	Advanced breeding line	-	University of Ghana
5	UG_05	Advanced breeding line	-	University of Ghana
6	UG_06	Advanced breeding line	-	University of Ghana
7	UG_07	Advanced breeding line	-	University of Ghana
8	UG_08	Advanced breeding line	-	University of Ghana
9	UG_09	Advanced breeding line	-	University of Ghana
10	UG_10	Advanced breeding line	-	University of Ghana
11	UG_11	Advanced breeding line	-	University of Ghana
12	UG_12	Advanced breeding line	-	University of Ghana
13	UG_13	Advanced breeding line	-	University of Ghana
14	UG_14	Advanced breeding line	-	University of Ghana
15	UG_15	Advanced breeding line	-	University of Ghana
16	UG_16	Advanced breeding line	-	University of Ghana
17	UG_17	Advanced breeding line	-	University of Ghana
18	UG_18	Advanced breeding line	-	University of Ghana
19	UG_19	Advanced breeding line	-	University of Ghana
20	UG_20	Advanced breeding line	-	University of Ghana
21	Apagbaala	Release variety (check)	2004	CSIR-SARI
22	Kirkhouse Benga	Release variety (check)	2016	CSIR-SARI
23	Wang Kae	Release variety (check)	2016	CSIR-SARI

### Experimental design and data collection

The experiments were laid out in a randomized complete block design (RCBD) with 3 replications in each location. Each genotype was assigned randomly. Seeds were planted on 4 m × 2.4 m (Area) plots having four rows, with inter and intra row spacing of 20 and 60 cm, respectively. The net harvest area was 4.8 m^2^ per plot, the central two rows. The space between plots was 1 meter and between blocks was 2 meters. Seedlings were thinned to two plants per hill at two weeks after planting. Planting was done within the first week of August in the Sudan savanna zone and the second week of September in the forest zones in both years. This coincides with the cowpea planting seasons at both locations. Weeding was done manually using hoes at 4 weeks after planting. The ‘scout and spray’ method was used for pest control. Insect pests were controlled by spraying a synthetic pyrethroid, lambda–cyhalothrin at a rate of 500 ml/ha. Pest control was at the vegetative, flowering and podding stages of the plants. No fertilizer was applied in this experiment to mimic farmer practice of cowpea production The data collected include days to 50% flowering which is the number of days from planting to when 50% of the plants in a plot flower. Days to maturity was recorded as the number of days from planting to when 90% of the pods in a plot reached physiological maturity. After harvesting, Pod length was recorded as the average of 10 randomly selected pods per plot measured from tip to tip. The number of seeds per pod was the average number of seeds from the 10 pods. Pod and grain weight per plot were recorded by weighing on a digital scale and used to compute yields in kg/ha. For each plot hundred seeds were counted weighed as hundred seed weight.

### Data analysis

Each location—year combination was regarded as an environment making 4 environments in the analysis. The combined analysis of variance ANOVA was done using a mixed model (genotype fixed and environment random) using GenStat 12^th^ Edition statistical software package to estimate the differences between genotypes across environments, among environments and their interaction. Additive and multiplicative interaction (AMMI) and GGE biplot were analyzed by using Genstat following the method outlined by Yan (2002) [15] to quantify genotype by environment interaction, classification of mega-environments and characterization of test environments and for simultaneous selection of genotypes based on stability and mean yield.

## Results and discussion

### Analysis of variance and estimation of variance components

The combined analysis of variance of grain yield (kg/ha) and yield-related traits of 23 cowpea genotypes tested in four environments is shown in Tables 4 and 5, respectively. The results showed that cowpea grain yield as well as yield related traits were highly significantly (*p* ≤0 .01) affected by environment, genotype, and genotype-environment interaction. These findings are in agreement with those of [16] who evaluated 25 cowpea landraces in six environments and observed that environment, genotype and genotype x environment interaction had significant effects on cowpea grain yield. Similarly, [17, 18] also reported that cowpea grain yield and yield-related traits were significantly affected by genotype, environment, and genotype by environment interaction. Environment and interaction contributed slightly to the total variation than genotype. This indicates that the genotypes did not perform consistently across the test environments. It could be hypothesized that reasons for the differences in the potentials of the genotypes across the test environments might be attributed to differences in such abiotic factors as soil, vegetation and rainfall; climatic differences across years may be a contributory factor.

**Table 4:** Combined ANOVA for grain yield (kg ha^-1^) of 23 cowpea genotypes tested in four environments.

Source of variation	Degrees of freedom	Sum of square	Mean square	Total variation explained
Genotype	22	17759563	807253***	0.28
Environment	3	18421781	6140594***	0.30
Rep (Env)	2	322790	161395	0.005
Interaction	66	19581180	296685***	0.31
Pooled error	182	6317854	34713	0.10
Total	275	62403169		

*** = Significant at 0.05, 0.001 and 0.001 probability level

**Table 5:** Mean square from combined ANOVA for yield related traits of 23 cowpea genotypes tested in four environments.

Source of variation	Df	DFF	DM	HSW	PL	SPP	SW	TPW	TSW
Genotype	22	50.81***	119.73***	60.25***	57.65***	34.58***	1.8***	299864***	112744***
Environment	3	552.41***	335.28***	64.05***	10.56***	1.42	2.6***	7656931***	2344315***
Rep (Env)	2	187.643	93.508	181.04	68.23	69.67	9.86	156299	57015
Interaction	66	5.72*	24.26***	3.91**	5.45***	6.44***	0.5***	129023***	48228***
Pooled error	182	3.777	2.62	2.34	1.04	1.24	0.2	12894	53772
Total	275								

Df = degree of freedom; DFF = days to 50% flowering; HSW = hundred seed weight; PL = pod length; SPP = Seeds per pod; SW = Seed Weight; TPW = total pod weight; TSW = total seed weight *,**, *** = Significant at 0.05, 0.001 and 0.001 probability level

The study has shown that in the current study, environments had different effects on the yield potentials of the 23 cowpea genotypes. This would therefore call for the need to do a further study on genotype-environment interaction to understand the nature of interaction and also to identify and select for stable genotypes.

### Additive main effects and multiplicative interaction (AMMI) analysis for grain yield

AMMI analysis of variance for grain yield (kg h^‐1^) of 23 cowpea genotypes tested in four environments are presented in Table 6. There was significant (*p* ≤ 0.01) effects of environment, genotype, and genotype-environment interaction. Main effects of environment, genotype, and genotype-environment interaction accounted for 33.04%, 31.85% and 35.12% of the total sum of squares, respectively in the analysis of combined variance. Total sum of squares of the model was attributed mostly to interaction and environmental effects indicating larger differences in genotypic responses across environments and the diverse those environments. This also designated the reliability of the multi-environment experiments. These findings are consistent with those of [16, 19] who worked with groundnut and cowpea, respectively and observed large contribution of GEI than environment and genotype effects for the observed yield variation. Multiplicative variance of treatment sum of squares was partitioned into three interaction principal components. These were all significant and explained 100.0% of the GEI. The first and second principal component axes (IPCA) of the interaction explained 51.35% and 32.23% of GEI sum of squares, respectively. [20] indicated that AMMI model with first and second multiplicative terms is adequate for cross validation of the variation in grain yield explained by GEI.

**Table 6:** Additive main effect and multiplicative interaction analysis of variance for grain yield (kg ha-1) of cowpea genotypes across four locations.

Source of variation	DF	SS	MS	Total variation explained	G x E explained
Environments (E)	3	18421781.49	6140593.83***	33.04	
Genotypes (G)	22	17759563.49	807252.89***	31.85	
Interaction (GxE)	66	19581180.26	296684.55***	35.12	
IPCA1	24	10152444.29	423018.51***		51.85
IPCA2	22	6310139.39	286824.52***		32.23
IPCA3	20	3118596.58	155929.83***		15.93
Residuals	184	6640643.76	36090.46***		

IPCA = Interaction principal component analysis

*** = Significant at 0.001 probability level

Among the testing environments, grain yields were highest at E2 with a mean grain yield of 1620.22 kg ha^−1^, the second highest of 1242.78 kg ha^-1^ was also recorded at E3, the third highest of 1111.83 kg ha^-1^ was recorded at E1 (Table 7). The lowest grain yield was obtained at E4 with a mean yield of 912.31 kg ha^−1^. The findings indicate that grain yields were higher at Bunso in 2022 and at Manga in 2021. However, at Bunso in 2021 and Manga in 2022 grain yields were lower. This could be attributed to changes in climatic conditions across years.

**Table 7:** Mean grain yield (kg ha^-1^) and genotype IPCA1 scores for 23 cowpea genotypes tested in four environments.

Genotypes	Environments				Genotype mean	IPCA1
	E1	E2	E3	E4		
1	1575	2215	1083	1172.9	1511	-13.19
2	1947	1812	981	738.1	1370	-15.23
3	468	879	795	290.5	608	4.67
4	1825	1595	2005	1098.6	1631	7.68
5	1230	1630	1540	1036.2	1359	4.44
6	368	1703	1135	1092.4	1075	4.69
7	1052	1761	1055	940.5	1202	-4.29
8	391	1312	1162	808.8	919	8.55
9	993	1328	1692	938.3	1238	12.52
10	799	1333	1096	698.5	982	2.95
11	760	1479	1013	776.1	1007	0.42
12	1338	1697	1564	1072.9	1418	3.18
13	880	1183	1743	880.7	1172	15.97
14	1497	1551	2143	1207.4	1600	14.05
15	990	2285	1145	1388.8	1452	-6.72
16	1049	1825	853	891	1154	-8.74
17	861	1477	1304	892.4	1134	5.03
18	1491	2127	1191	1178.5	1497	-9.46
19	1375	1653	851	687.4	1142	-10.51
20	470	1091	880	489.1	733	4.35
21	1591	1780	939	775.4	1271	-12.13
22	1361	1824	1011	895.1	1273	-8.85
23	1261	1725	1403	1033.6	1356	0.62
Mean	1111.83	1620.22	1242.78	912.31	1221.91	
Env IPCA1	-17.50	-20.38	33.09	4.79		

The sign of IPCA scores is an indication of the pattern of interaction of genotypes across environments and vice versa. Genotypes and environments with a similar sign of their IPCA scores interact positively for that trait [16]. In the current study environments E1 and E2 differed from the other two environments in both interaction and main effects. They both had a negative IPCA1 score. Environment E1 had mean grain yield below the grand mean while E2 had a mean grain yield above the grand mean. Environments E3 and E4 showed positive environment IPCA1 scores with mean grain yield above and below the grand mean, respectively.

With reference to absolute values of IPCA scores, genotypes with large scores have high interactions indicating that they are not stable, while genotypes with small IPCA1 scores close to zero have small interactions and indicate that they are stable [21]. Therefore, in the current study genotypes 11, 23, 10 and 12 obtained relatively smaller IPCA1 scores indicating that they can be considered to be stable with a wider adaptation. Genotypes 13, 2, 14, 1, 9 and 21 had relatively higher IPCA1 scores. Similarly, environments that show relatively higher IPCA scores discriminate among genotypes more than environments with relatively lesser scores [16]. In the current study, environment E3 was the most discriminating environment for the genotypes followed by E2.

Genotype 4 was one of the four best genotypes selected by the AMMI model. It was selected as the 2^nd^ best genotype in two different environments (Table 8). Genotype 4 was selected in environment E1 (unfavorable environment), where the environmental mean yield was lower than the grand mean, and in environment E3 (favorable environment), where the environmental mean yield exceeded the grand mean. Thus, this genotype is suitable for cultivation in both environments.

**Table 8:** The first four best cowpea genotypes selected for mean yield by AMMI model per environment.

Environment	Mean (kg ha^-1^)	IPCA score	1	2	3	4
E1	1112	-17.5	G2	G4	G21	G1
E2	1620	-20.38	G15	G1	G18	G16
E3	1243	33.09	G14	G4	G13	G9
E4	912	4.79	G15	G14	G18	G1

E1 = Bunso 2021, E2 = Bunso 2022, E3 = Manga 2021, E4 = Manga 2022

Genotype 14 (the second highest yielding genotype) was also selected at two different environments E3 and E4 which were favorable and unfavorable, respectively. The third-highest yielding genotype was genotype one (1) which was selected in two different environments E1 and E4 (both are unfavorable). The fourth-highest yielding genotype was 18 which was selected at environments E2 (a favorable environment) and E4 (an unfavorable environment). From AMMI’s best four selections, genotypes 4, 14, 18 and 15 were suitable in both favorable and unfavorable environments, however genotypes 9, 13, 16 and 21 were suitable in favorable environments. Genotypes 1 and 2 were suitable in unfavorable environments. Selection of these genotypes by AMMI model in their respective environments is evident of the best adaptation of the genotypes in those environments. These findings are consistent with those made by [16] for cowpea. They observed cowpea genotypes that were desirable in both favorable and unfavorable environments, they also observed genotypes that were desirable only in either favorable environment or unfavorable environment.

### Stability of the genotypes

**Fig 1:**
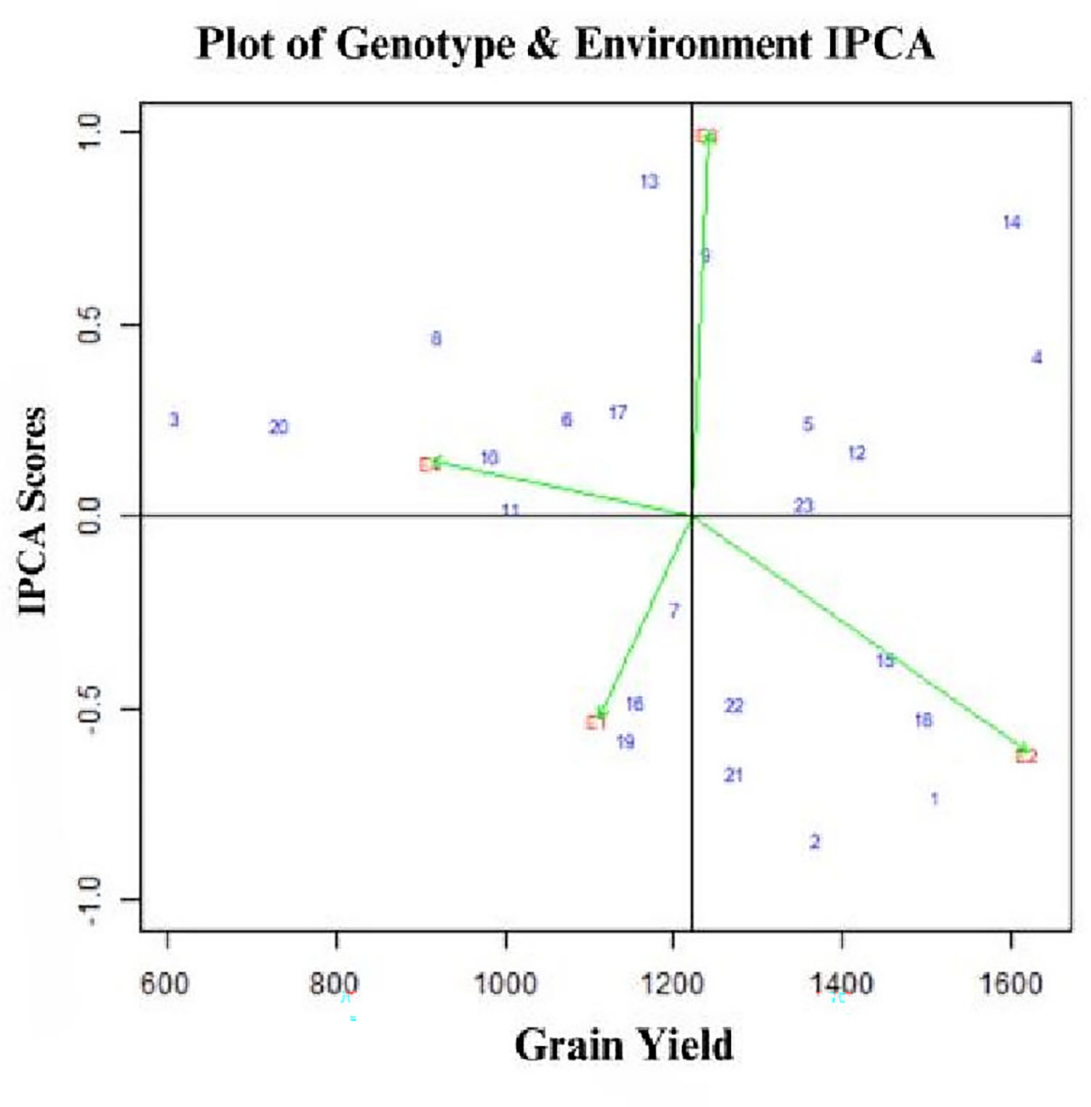
AMMI biplot for PCA1 scores and grain yield of 23 cowpea genotypes. E1 = Bunso 2021, E2 = Bunso 2022, E3 = Manga 2021, Manga 2022, See genotype codes in Table 1.

In the two-dimensional AMMI biplot, x-axis represents the genotypes and environment main effect and y-axis represents the interaction effects [22]. Genotypes located near the x-axis to the right of y-axis are considered as stable and high-yielding, whereas genotypes located far from x-axis to the left side of y-axis are considered as unstable and low-yielding (Fig 1). Accordingly, genotypes 11 and 10 are stable but low-yielding, while genotypes 23 and 12 are stable and high yielding and may be less influenced by environment. Genotype 14 is the highest-yielding but the most unstable with the highest IPCA1 score. Generally, superior genotypes with regard to grain yield, were genotypes 4, 14, 23 and 12.

Environment 3 (E3) showed high IPCA scores and contributed largely to genotype-environment interaction. E3 was the most favorable environment for high yielding genotypes which had higher yield means than the grand mean. E4 was the least favourable environment for all the genotypes with low yield and lower IPCA1 scores. Stable genotypes are characterized by low IPAC1 scores and therefore genotypes UG_23, UG_11, UG_12, UG_7 and UG_04 were stable and therefore less influenced by environments.

### Ideal genotype

**Fig 2:**
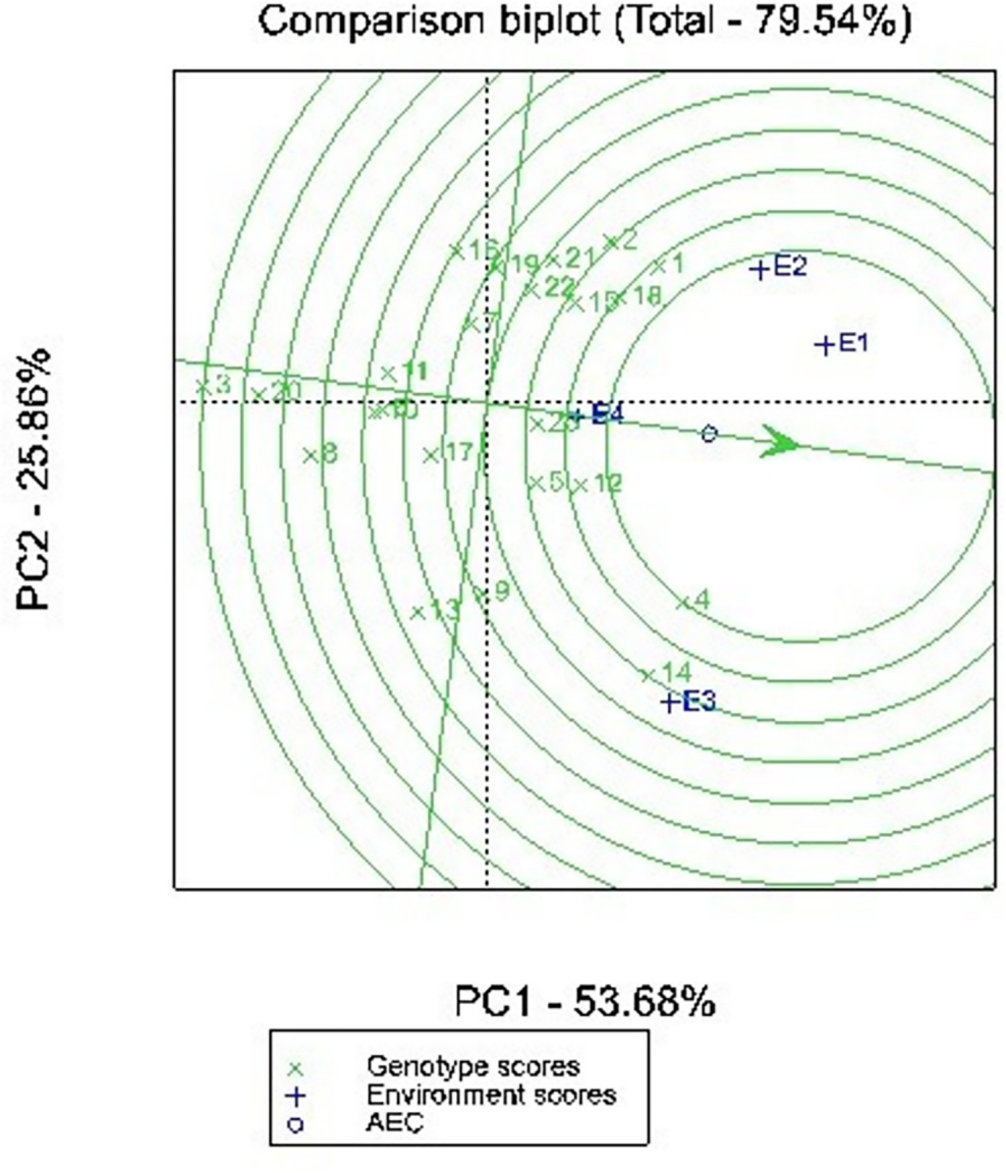
GGE biplot showing comparison of the test genotypes with the ideal genotype based on mean grain yield of 23 cowpea genotypes across 4 environments. E1 = Bunso 2021, E2 = Bunso 2022, E3 = Manga 2021, E4 = Manga 2022, See genotype codes in Table 3.

The GGE biplot methodology [11, 15, 23] consists of a set of biplots interpretation methods, whereby important questions regarding genotype evaluation and test-environment evaluation can be visually addressed. The results of the GGE biplot revealed that the first two principal components explained 79.54% of the total yield variation across the tested environments (Fig 2). PCA1 and PCA2 explained 53.68% and 25.86%, repectively. This results was higher than what was obtained by [14] who reported in their study the first and second princiapl component accounted for a total of 69.59% of the yield variation observed. Identification of the ideal genotype is illustrated in Fig 2. The small circle in Fig 2 which is located on the average environment coordinate (AEC) and with an arrow pointing to it, represents the ideal genotype. The ideal genotype is defined by the two criteria: (i) it has the highest yield of the entire dataset, and (ii) it is absolutely stable since it is located on AEC abscissa [24]. The genotypes can therefore be ranked by performance based on their distance from the ideal cultivar. Genotype UG_04 was closest to the ideal genotype and therefore most desirable of all the tested genotypes. It was followed by genotypes UG_01, UG_12, and UG_18. In contrast, genotypes UG_03, UG_08 and UG_20 were farthest from the ideal gentoype, signifying their low yield and poor stability.

### The “which-won-where” analysis the which won where and what GGE biplot

**Fig 3:**
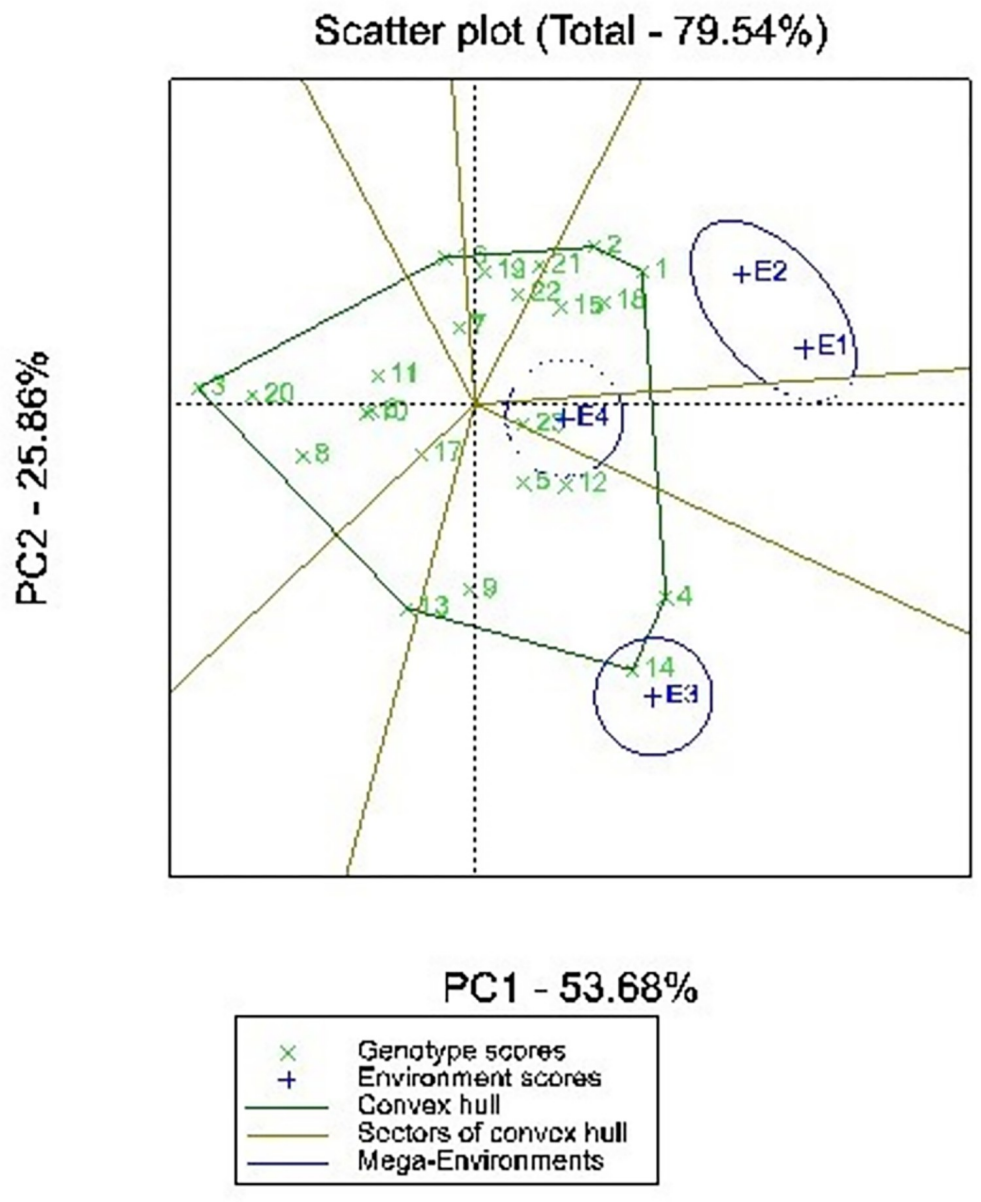
Polygon view of GGE biplot showing which genotype performed best in which environments based on mean grain yield of 23 cowpea genotypes across 4 environments. E1 = Bunso 2021, E2 = Bunso 2022, E3 = Manga 2021, E4 = Manga 2022, See genotype codes in Table 3.

A polygon view of a GGE biplot provides an effective means of viewing the “which-won-where” pattern of multi-environmental trial dataset [24]. The polygon is drawn by joining the genotypes that are farthest from the origin of the biplot whereby all other genotypes are contained in the polygon. The biplot is divided into sectors by drawing perpendicular lines to each side of the polygon. The genotype in each sector is the highest-yielding genotype in environments that fall in the particular sector. Fig 3 shows the polygon view of a GGE biplot showing the best performing genotype in which environment. The biplot classified the tested environments into 3 mega environments. Environments E1 and E2 formed one mega-environment, environment E3 formed a second mega-environment, while environment E4 formed a third meg-environment. The best performing genotypes in mega-environments E1 and E2 was UG_01. The best performing genotypes in mega-environment E3 was UG_14.

Genotypes located at the vertex of the polygon without any environment in that sector are considered as poorly performed genotypes at all the tested environment and cannot be selected for grain yield improvement [25, 26]. Similarly, genotypes placed within the polygon were considered to be less responsive.

### Ideal environment

**Fig 4:**
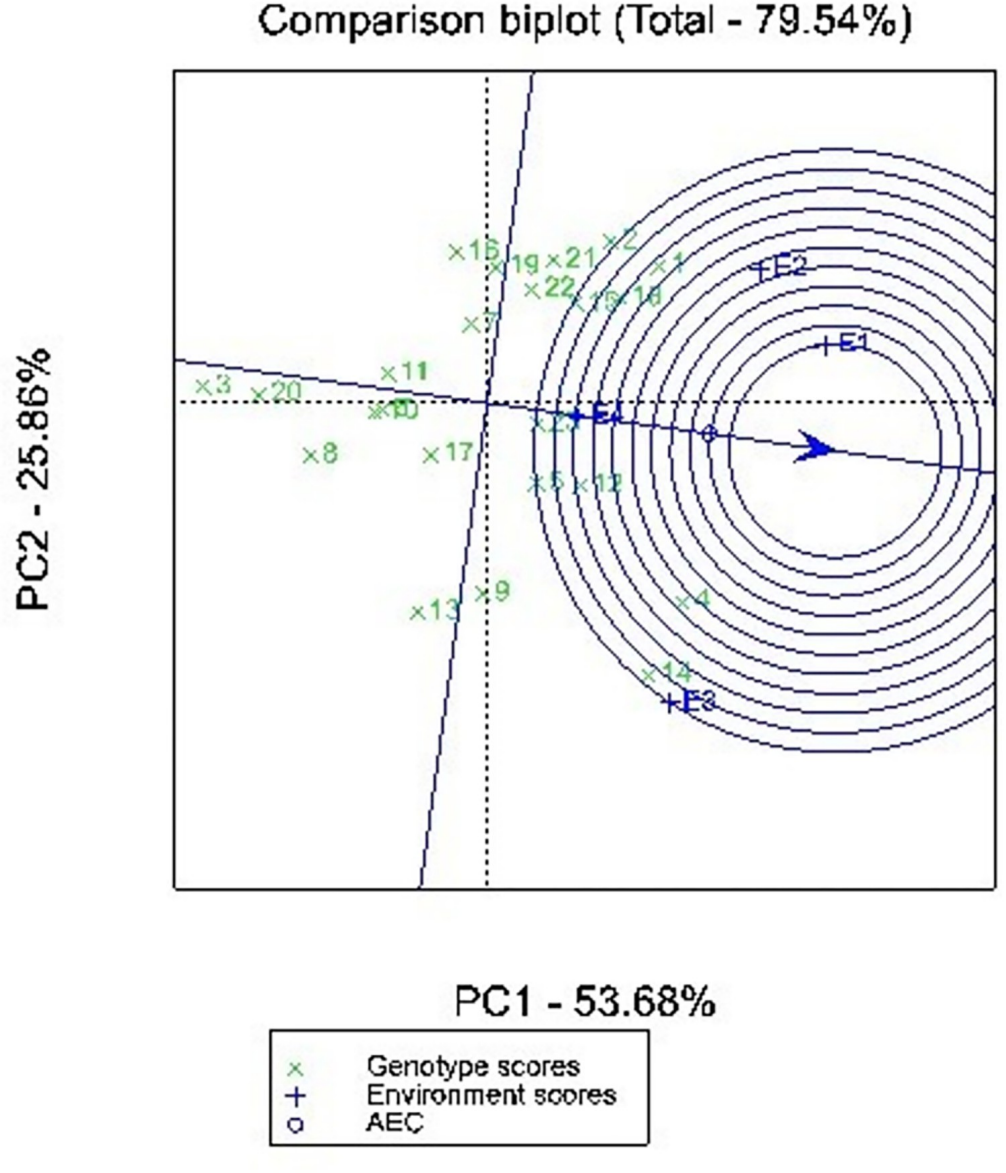
GGE biplot showing comparison of 4 environments with the ideal environment based on mean grain yield of 23 cowpea genotypes across 4 environments. E1 = Bunso 2021, E2 = Bunso 2022, E3 = Manga 2021, Manga 2022, See genotype codes in Table 3.

Test environment evaluation is designed to identify the environments that can effectively identify superior genotypes for mega-environments. One key feature of the GGE biplots is its ability to help identify the ideal environment for distinguishing the tested genotypes and the ability of that environment to represent all the evaluated environments [27]. This is very crucial in the selection of superior genotypes from the tested genotypes across the tested environments. The ideal environment is located at the center of a set of concentric lines of the GGE biplot. The Centre serves as a reference point to measure the distance between an environment and the ideal environment. The environments are therefore ranked based on their distance from the ideal environment. Fig 4 illustrates comparison of all the four environments with the ideal environment. Environment E1 is identified as the ideal environment since it is closest to the center of the set of concentric lines. Hence planting in environment E1 (Bunso_2021) provided the most ideal environmental conditions. Moreover, this environment allowed all the tested genotypes to express their genetic potential and provides extra discriminatory ability to complement the other environments. On the contrary, environment E3 (Manga_2022) was identified as the worst environment and suitable for selecting superior genotypes for grain yield in cowpea.

## Conclusion

The Study revealed significant variation in grain yield attributable to genotype, environment and their interaction. Notably, the interaction between genotype and environment (GEI) accounted for a substantial portion of the total variation in grain yield. Specifically, the first two principal components, IPAC1 and IPCA2 explained 84.08% of the total GEI, indicating that these principal components effectively captured the interaction effects among the tested genotypes. Using the GGE biplot analysis, the environments were classified into three distinct mega-environments. Bunso_2021 and Bunso_2022 were grouped into one mega-environment, while Manga_2021 and Manga_2022 were categorized into separate mega-environments. Among these, the Bunso_2021 environment emerged as the most ideal, as it allowed all tested genotypes to fully express their genetic potential and provided enhanced discriminatory ability compared to other environments. Therefore, Bunso_2021 is recommended for selecting superior genotypes for specific agro-ecological zones in Ghana.

Additionally, genotype UG_04 demonstrated higher yield and stability across all the tested environments, outperforming other genotypes. This suggests that UG_04 possesses exceptional adaptability and consistent performance, making it a promising candidate for cultivation in diverse conditions.

## Supporting information

S1 File Raw data behind the means in Table 7. (XLSX)
